# Diagnostic Challenges in Pulmonary Embolism in Young Adults: Thrombosis Associated With Cytomegalovirus and Mycoplasma pneumoniae

**DOI:** 10.7759/cureus.32757

**Published:** 2022-12-20

**Authors:** Haruka Hikichi, Ryo Hasegawa, Akiko Saga, Tomoo Saga, Shigeharu Ueki

**Affiliations:** 1 Department of General Internal Medicine and Clinical Laboratory Medicine, Akita University Graduate School of Medicine, Akita, JPN

**Keywords:** lupus anticoagulant, mycoplasma pneumoniae infection, cytomegalovirus (cmv), splenic infarction, pulmonary embolism (pe)

## Abstract

A 23-year-old man presented with a fever, shaking chills, headaches, nausea, and a dry cough. Investigations showed lymphocytic leukocytosis with atypical lymphocytes in a blood smear. Liver function test results, D-dimer concentrations, and fibrin degradation product concentrations were greatly elevated. Computed tomography of the whole body with contrast showed hepatosplenomegaly with splenic infarction and bilateral pulmonary embolism without deep vein thrombosis. Cytomegalovirus (CMV) immunoglobulin M, and serum CMV pp65 antigenemia were positive, and serum *Mycoplasma pneumoniae* (*M. pneumoniae*) antibody was also highly positive. These results suggested the diagnosis of co-infection of CMV and *M. pneumoniae* complicated by systemic arteriovenous thrombosis, which resulted in pulmonary embolism and splenic infarction. After he started edoxaban tosilate hydrate for the thrombosis, his symptoms resolved in a few days. To the best of our knowledge, this is the first case of co-infection of CMV and *M. pneumoniae* leading to pulmonary embolism and splenic infarction.

## Introduction

Although an acute cytomegalovirus (CMV) infection in immunocompetent patients is generally asymptomatic or self-limiting, CMV associated with vascular thrombosis, including deep vein thrombosis, thrombophlebitis, splenic infarction, and pulmonary embolism has been increasingly recognized. There have been more than 100 case reports, three retrospective studies, two prospective studies, and one cohort trial on this condition. A case-control study showed that 6.4% of 140 patients with acute CMV infection had thrombosis, 3.6% of them had arterial thrombosis, and 2.9% of them had venous thrombosis [1]. Another study showed that 65.9% of 64 patients were immunocompetent [2].

Even though the occurrence of multipathogen infections in children with Epstein-Barr virus (EBV)/CMV primary infection is more than 60% [3], co-infection of EBV/CMV and other agents in adults has not been well reported. Li et al. reported a case of a 19-year-old woman with co-infection of EBV, CMV, and* Mycoplasma pneumoniae* (*M. pneumoniae*) associated with splenic infarction [4]. To the best of our knowledge, the present report is the first of pulmonary embolism and splenic infarction associated with the co-infection of CMV and *M. pneumoniae*.

## Case presentation

A 23-year-old man presented to a general physician with a four-week history of a persistent fever with shaking chills, headaches, nausea, and a dry cough. He had no confusion, stiff neck, seizures, chest or abdominal pain, or skin rash. His medical history included a panic disorder, which was well controlled with anti-depressants and benzodiazepines. He smoked six cigarettes each day. He denied drinking alcohol or using recreational drugs.

On examination, he was tachycardic with a heart rate of 150 beats/minute but normotensive with a blood pressure of 129/90 mm Hg. His temperature was 36.8°C, his respiratory rate was 12/min, and his oxygen saturation was 98% in room air. His Glasgow Coma Scale score was 15. Jolt accentuation of the headache and neck stiffness were negative. Neither tonsils nor lymph nodes were swollen. Cardiac and respiratory examinations were normal, except for tachycardia. He had no abdominal tenderness. There were no abnormal findings of the skin or the joints.

Laboratory findings (Table 1) showed that the hemoglobin concentration was 13.3 g/dL, platelet count was 358,000 cells/µL, and leukocyte count was 9800 cells/µL (47% of the cells were lymphocytes, 3% were atypical lymphocytes, and 44% were neutrophils). The aspartate aminotransferase concentration was 50 IU/L, alanine transferase concentration was 63 IU/L, alkaline phosphatase concentration was 137 IU/L, γ-glutamyl transferase concentration was 226 IU/L, lactate dehydrogenase concentration was 451 IU/L, serum copper concentration was 226 µg/dL, and ferritin concentration was 691.1 µg/L. The C-reactive protein concentration was 4.25 mg/L, and the erythrocyte sedimentation rate was 38 mm/h. The D-dimer concentration was 14.01 µg/mL and the fibrin degradation product (FDP) concentration was 28.9 µg/mL. Human immunodeficiency virus types 1 and 2 antigens and antibodies were negative. Two sets of blood cultures and a cerebrospinal fluid culture were negative. An electrocardiogram showed sinus tachycardia. A chest radiograph showed no abnormalities in the heart or the lungs.

**Table 1:** Laboratory findings of the patient. FDP: fibrin-fibrinogen degradation product; Anti-cardiolipin β2GPI antibody: anti-cardiolipin β2-glycoprotein I complex antibody; CMV: cytomegalovirus; IgM: immunoglobulin M; IgG: immunoglobulin G; *M. pneumoniae*: *Mycoplasma pneumoniae*; PA: particle agglutination; EBV: Epstein-Barr virus; EBNA: Epstein-Barr virus nuclear antigen; HIV: human immunodeficiency virus;

Laboratory findings	Patient value visited				Reference range
	Day 0	Day 2	Day 9	Day 30	
Hemoglobin (g/L)	13.3	12.4	12.9	14.1	13.5-18.5
Platelet count (cells/µL)	358000	346000	440000	288000	130000-370000
White cell count (cells/µL)	9800	9800	9800	9100	3900-9800
Neutrophils (%)	44	47.4	44	55.2	38-58
Lymphocytes (%)	47	41.5	37	33.7	20-60
Atypical lymphocytes (%)	3	0	0	0	-
Aspartame aminotransferase (IU/l)	50	51	52	58	8-38
Alanine transaminase (IU/l)	63	59	73	105	4-43
Alkaline phosphatase (IU/l)	137	141	130	81	38-113
γ-glutamyl transferase (IU/l)	226	221	164	101	≦86
Lactate dehydrogenase (IU/l)	451	360	289	226	120-442
Copper (µg/dL)	226	216	193	208	70-132
Ferritin (µg/L)	691.1				17.0-291.5
C-reactive protein (mg/L)	4.25	4.54	0.21	0.15	≦0.3
Coagulation molecular workup					
D-dimer (µg/mL)	14.01	12.88	5.56	0.74	≦1.0
FDP (µg/mL)	28.9	26.8			≦4.0
Antithrombin III		117.8		>150	79-121
Protein C activity (%)		100.8		>150	64-145
Antinuclear antibody	×80				＜×40
Lupus anticoagulant	1.25 (positive)				≦1.16
Anti-cardiolipin β2GPI antibody (U/ml)	<1.2 (negative)				<1.2
Anti-cardiolipin antibody (U/ml)	8.4 (negative)				≦12.3
Infections					
CMV IgM index	13.51 (positive)			3.42 (positive)	<0.85
CMV IgG titre	83.2 (positive)			137 (positive)	<6.0
CMV antigenemia			Positive	Negative	-
*M. pneumoniae* antibody (PA)		1280 (positive)		320 (positive)	<40
EBV IgM	<10 (negative)				<10
EBV IgG	640 (positive)				<0.5
EBNA	160				<10
HIV-1 and HIV-2 antigen / antibody	Negative			Negative	-
COVID-19 nasopharyngeal PCR	Negative				-
Hepatitis B virus antigen	Negative				-
Hepatitis C virus antibody	Negative				-

**Figure 1:**
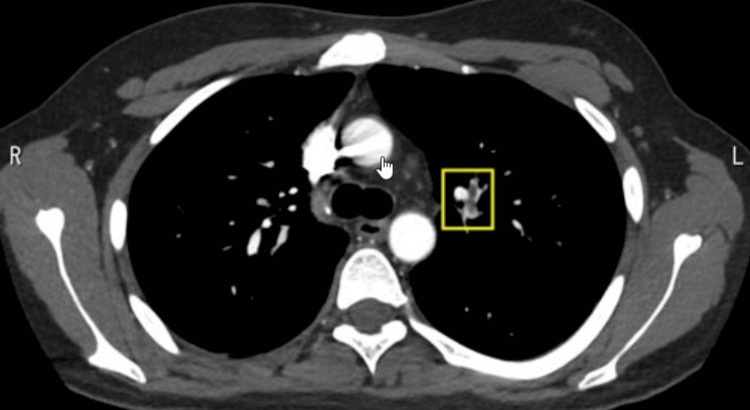
Initial imaging study of the chest An enhanced CT image of the chest on initial presentation shows left pulmonary embolisms (framed areas).

**Figure 2:**
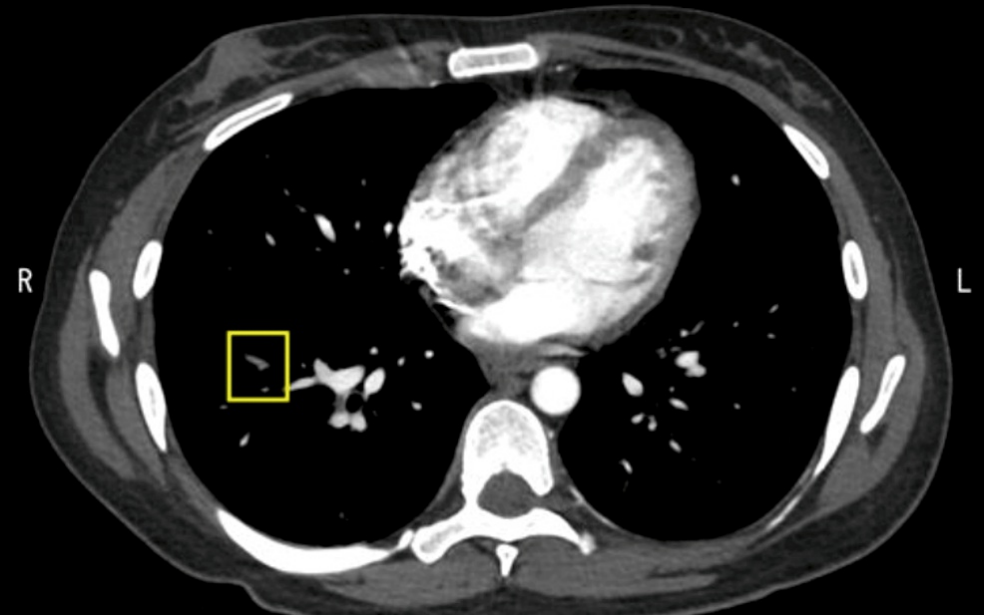
Initial imaging study of the chest An enhanced CT image of the chest on initial presentation shows right pulmonary embolisms (framed areas).

**Figure 3:**
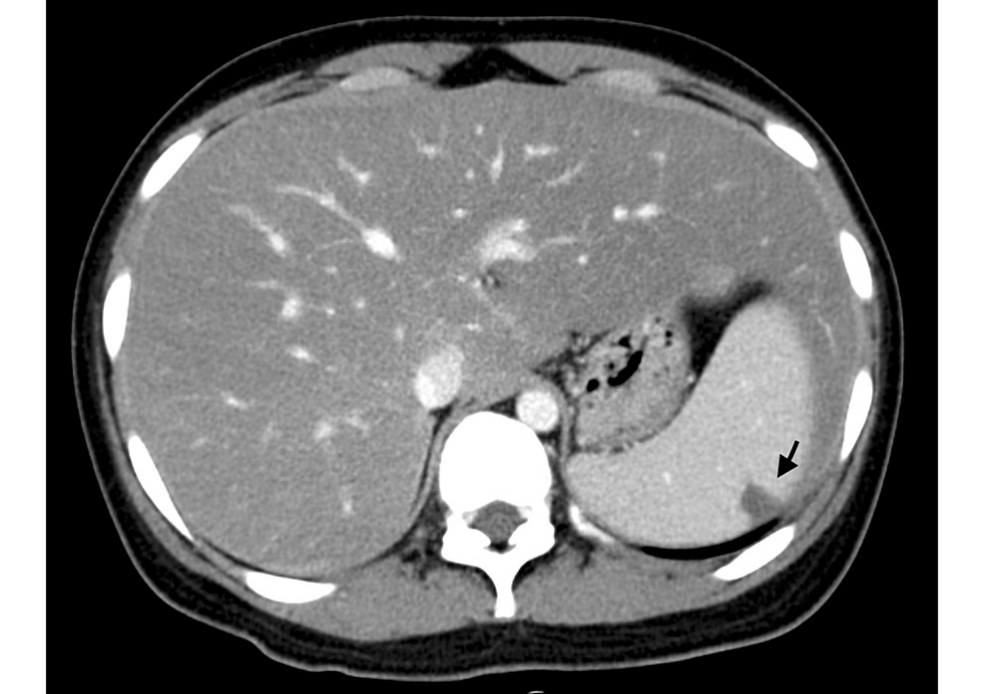
Initial imaging study of the abdomen An initial enhanced CT image of the abdomen shows hepatosplenomegaly and a splenic infarction (arrow).

Chest computed tomography (CT) revealed bilateral pulmonary embolism without consolidation (Figure 1, 2). CT of the abdomen (Figure 3) demonstrated hepatosplenomegaly and splenic infarction. CT of the lower limbs showed no deep vein thrombosis. The CMV immunoglobulin (Ig) M index was 13.51 (reference range, <0.85), CMV IgG was 83.2 arbitrary units/mL (normal range, <6.0), and serum CMV pp65 antigenemia assay was at least one positive. The anti-*M. pneumoniae* IgM titer was 1280 (reference range, <40). Epstein-Barr virus viral capsid antigen IgM titer was <10 (reference range, <10), and the viral capsid antigen IgG titer was 640 (reference range, <0.5). The EBV nuclear antigen (EBNA) antibody titer was 160 (reference range, <10). A coronavirus disease 2019 nasopharyngeal polymerase chain reaction test was negative, hepatitis B surface antigen was negative, and hepatitis C virus antibody was negative. Lupus anticoagulant was 1.25 (reference range, <1.20), anti-cardiolipin β2-glycoprotein I antibody was <1.2 U/mL (negative), and anti-cardiolipin antibody was 8.4 U/mL (negative).

Finally, this case showed the complication of pulmonary embolism and splenic infarction (i.e., arteriovenous thrombosis). Serological tests indicated acute co-infection of CMV and *M. pneumoniae*. CMV and *M. pneumoniae* can contribute to transient positivity for lupus anticoagulants. These results suggested the diagnosis of co-infection of CMV and *M. pneumoniae* complicated by systemic arteriovenous thrombosis, which resulted in pulmonary embolism and splenic infarction.

After the patient started 30 mg of edoxaban tosylate hydrate for thrombosis, all of his symptoms, including the fever and headaches, resolved in a few days without antiviral medications and antibiotics. A serological test one month later showed that the CMV IgM index was 3.42 (decreased), CMV IgG titer was 137 (increased), and serum CMV pp65 antigenemia was negative. *M. pneumoniae* antibody was 320 (decreased). A follow-up chest CT one month after the patient’s initial presentation revealed resolution of the pulmonary embolism. He completed anticoagulation therapy for four months.

## Discussion

CMV infection interferes with coagulation pathways, resulting in thrombosis. CMV activates factor X and systemic endotheliitis, and increased tissue factor expression occurs. The production of factor VIII and von Willebrand factor are also increased. These mechanisms cause platelet and leukocyte aggregation, adhesion, and thrombin formation [5]. CMV is also a causal factor inducing antiphospholipid antibody syndrome with associated vascular thrombosis [6].

*M. pneumoniae* is associated with secondary thrombosis [7]. Previous case reports showed that pulmonary embolism [8] or splenic infarction [9] with *M. pneumoniae* infection occurred even without pneumonia. Although the mechanism of extrapulmonary invasion of *M. pneumoniae* is poorly understood, direct invasion to a secondary organ, immunological damage, or vascular obstruction is possible. Furthermore, a prospective study of United Kingdom women showed that the use of antidepressant drugs was related to a high risk of venous thromboembolism [10]. Our patient also had a risk of thrombosis because of the long-term use of antidepressants.

D-dimer and FDP are typically chosen for the screening of thrombosis. There are no screening guidelines for patients with acute CMV infection and/or *M. pneumoniae*. Lower-extremity deep vein thrombosis and/or venous thromboembolism leading to splanchnic vein thrombosis, including the portal vein, superior mesenteric vein, inferior mesenteric vein, and colic vein are common thromboses related to CMV infections [2]. Therefore, screening with lower-extremity Doppler is cost-effective [11]. Chest and abdominal CT scans with contrast are recommended for a more accurate diagnosis of pulmonary embolism, portal vein thrombosis, and splenic infarction. A retrospective study of 43 children with *M. pneumoniae* associated with thrombosis suggested that 25 (58.1%) patients showed D-dimer concentrations >5.0 mg/L, and the mean D-dimer concentration was 11.1 ± 12.4 mg/L [12].

## Conclusions

Physicians should be aware of thrombosis complicated by common infectious diseases, such as CMV and *M. pneumoniae*, even in immunocompetent young adults. A contrast-enhanced computed tomography scan, echocardiography, and blood vessel ultrasonography are recommended in the case of elevated D-dimer concentrations to detect thrombosis, including pulmonary embolism and splenic infarction.
